# Phage Φ170 biocontrol of multidrug-resistant *Vibrio parahaemolyticus* in a zebrafish model

**DOI:** 10.1128/spectrum.00089-26

**Published:** 2026-05-26

**Authors:** Wanqiu Lin, Linlin Ye, Xuhang Wang, Jianluan Ren, Feng Xue, Jianjun Dai, Fang Tang

**Affiliations:** 1 MOE Joint International Research Laboratory of Animal Health and Food Safety, Key Laboratory of Animal Bacteriology, Ministry of Agriculture, College of Veterinary Medicine, Nanjing Agricultural University 718390 , Nanjing, China; 2 School of Life Science and Technology, China Pharmaceutical University 56651, Nanjing, China; Northwest A&F University College of Animal Science and Technology, Yangling, Shaanxi, China

**Keywords:** *Vibrio parahaemolyticus* phage, multidrug-resistant *Vibrio parahaemolyticus*, zebrafish model, phage prevention and treatment

## Abstract

*Vibrio parahaemolyticus* is an important zoonotic pathogen that poses a persistent threat to aquaculture systems and food safety. The rapid emergence of multidrug resistance, driven by increasing antibiotic selection pressure, highlights the urgent need for alternative antimicrobial strategies, among which phage therapy has emerged as a promising candidate. Here, we reported the isolation of a bacteriophage, designated Φ170, targeting multidrug-resistant *Vibrio parahaemolyticus* from sewage collected at a shrimp farm. Φ170 exhibited a latent period of 10 min, an eclipse period of approximately 60 min, and an average burst size of 426 PFU per cell. The phage also showed broad tolerance to pH and temperature. Φ170 displayed rapid and potent lytic activity, effectively suppressing host bacterial growth within 6 h post-infection. To further assess its in *vivo* efficacy, we evaluated the prophylactic and therapeutic potential of phage administration by immersion in zebrafish challenged with *Vibrio parahaemolyticus*. Prophylactic treatment with Φ170 significantly increased zebrafish survival within 72 h. Moreover, combined treatment with Φ170 and doxycycline exerted a synergistic effect, effectively reducing bacterial loads, alleviating liver and intestine tissue damage, and suppressing excessive inflammatory responses. Collectively, these findings indicated that phage Φ170 represented a promising prophylactic agent against *Vibrio parahaemolyticus* infection and that combined phage therapy with antibiotics provided an effective strategy for treating drug-resistant *Vibrio parahaemolyticus* infections.

**IMPORTANCE:**

Phage therapy is a promising strategy for combating bacterial infections, serving as a complementary therapy to antibiotics and a synergistic approach in combination treatments with antimicrobial agents. To address infections by multidrug-resistant (MDR) *Vibrio parahaemolyticus*, we isolated and characterized bacteriophage Φ170, which is capable of lysing MDR *Vibrio parahaemolyticus*. We evaluated its prophylactic and therapeutic potential in a zebrafish infection model. We found that phage Φ170 exhibited strong lytic activity both *in vitro* and *in vivo*, effectively preventing *Vibrio parahaemolyticus* infection and displaying synergistic antibacterial effects when combined with antibiotics. Phage-antibiotic combination therapy further enhanced therapeutic efficacy in infected zebrafish. Our results provide experimental support for phage-antibiotic combination strategies against MDR bacterial infections and highlight the potential of phage therapy for the prevention and treatment of *Vibrio parahaemolyticus* infections.

## INTRODUCTION

*Vibrio parahaemolyticus* (Vp) is a significant zoonotic pathogen that is widely distributed in coastal, estuarine, and various aquatic environments worldwide (1). This pathogen can infect a broad range of aquatic organisms, including fish, shrimp, and shellfish, causing severe diseases and substantial economic losses in aquaculture industries (2). Vp is primarily the most prevalent coastal pathogen associated with seafood consumption. In humans, consumption of food contaminated with Vp can lead to foodborne illnesses such as diarrhea and gastroenteritis (3). Outbreaks caused by Vp occur frequently and have increased in recent years, posing a persistent threat to food safety and public health (4). Currently, the prevention and control of bacterial diseases in aquaculture still rely largely on antibiotics. Although regulatory measures on antibiotic use have been strengthened in recent years, the overuse of antibiotics in aquaculture has driven the emergence of antimicrobial resistance in many pathogens, including Vp, and has contributed to the spread of multidrug-resistant (MDR) strains (5). Therefore, there is an urgent need to develop alternative strategies to replace antibiotics and effectively control MDR Vp infections in aquaculture systems.

Phages are viruses that exhibit high host specificity and are widely distributed in natural environments. Even at the time of their initial discovery, the potential of phage therapy for preventing and treating bacterial diseases was recognized, and early studies demonstrated promising results in both animal models and clinical settings (6, 7). However, the widespread adoption of broad-spectrum antibiotics in modern medicine curtailed the development and clinical application of phage therapy in Western countries, while its use and refinement continued in several Eastern European countries, partly due to limited access to antibiotics (8). In recent years, with the global rise of antibiotic resistance, phage therapy has regained attention as an alternative to antibiotics and as an effective approach for controlling foodborne pathogens (9). Phage therapy offers several distinct advantages. Unlike antibiotics, phages exhibit high host specificity, targeting only specific bacterial strains without disrupting the host’s commensal microbiota (10). Phages are not affected by conventional mechanisms of bacterial drug resistance, even MDR bacteria or “superbugs” remain susceptible to phage-mediated lysis (11). Moreover, phages possess the ability to self-replicate, allowing continued amplification as long as susceptible host bacteria are present, thereby enhancing bacterial clearance. Although endotoxins released during phage-mediated bacterial lysis may trigger inflammatory responses, current evidence suggests that such effects are minimal (12). Phages demonstrate excellent safety, supporting their potential application in clinical phage therapy.

In recent years, phage therapy has emerged as a promising approach for combating infections caused by bacteria (9, 13). A growing body of evidence from animal models and preclinical studies has demonstrated that phages not only exhibit inhibitory activity against MDR pathogens but also display potent lytic activity against susceptible strains (14, 15). The application of phages in infections caused by susceptible bacteria may help reduce antibiotic usage, thereby mitigating the emergence and transmission risk of MDR strains at the source (16, 17). In the field of aquaculture, phage therapy has similarly shown considerable potential for the prevention and control of major bacterial pathogens, particularly *vibrio* species (18). Multiple phages targeting Vp have been isolated and evaluated in experimental infection models. For instance, a phage cocktail composed of PVP1 and PVP2 significantly improved the survival of sea cucumbers infected with Vp (19), and phage VPp1 reduced the bacterial loads in oysters by 2–3 orders of magnitude within 36 h (20). However, reports describing phages that specifically target MDR Vp remain limited (21). Although substantial progress has been made in the development of phage therapy, several challenges still hinder its practical application, including environmental complexity, phage selection, and optimization of administration strategies (22). Therefore, the isolation and characterization of phages with high stability, strong replication capacity, and favorable safety profiles are essential for successful application. In aquaculture settings, oral administration and immersion are the two most commonly used delivery routes (23). Jun et al. demonstrated that prophylactic administration of phage pVp-1 via oral feeding or immersion increased the survival of *Litopenaeus vannamei* following Vp infection by 50% and 75%, respectively, highlighting the potential advantage of immersion treatment during early stages of infection (24). However, the optimal application parameters for different phages still require further experimental validation. Moreover, strategies, such as the development of phage cocktails or the combination of phages with antibiotics, have been proposed to further enhance therapeutic efficacy against MDR Vp (25, 26). Collectively, these advances underscore the potential of phage-based interventions as a viable alternative for controlling MDR *Vibrio* infections in aquaculture.

In this study, a phage Φ170, capable of lysing MDR Vp, was isolated from the sewage of a shrimp farm in China. The phage was administered directly to aquaculture water using an immersion-based approach, and its prophylactic effect against infection with the MDR strain Vp94, as well as its therapeutic potential in combination with antibiotics, was evaluated in a zebrafish model. This study provides evidence supporting the potential application of phage therapy for the prevention and treatment of Vp infections.

## MATERIALS AND METHODS

### Bacterial strains and growth conditions

The MDR *Vibrio parahaemolyticus* strain Vp94 and other *Vibrio* strains used in this study were isolated from shrimp larvae collected from aquaculture farms. Samples were streaked on Thiosulfate-Citrate-Bile Salts-Sucrose (TCBS) agar plates and incubated at 37°C overnight. Single colonies were selected and inoculated into LB broth supplemented with 3% NaCl, followed by incubation at 37°C with shaking. The identification of the strains was confirmed by PCR amplification of the *16S rRNA* (F: AGAGTTTGATCCTGGCTCAG, R: GGTTACCTTGTTACGACTT) (27) and *HSP60* (F: ACAACAGCAACGGTACTAGC, R: CAACTTTCACGATGCCAC) (28) genes. All strains were stored at −80°C in LB broth supplemented with 20% glycerol.

### Assay 1—phage isolation and purification

Sewage samples collected from a shrimp farm in Jiangsu Province in China were centrifuged at 8,000 rpm for 10 min to remove impurities, then filtered through a 0.22-μm microporous membrane to remove any remaining bacteria. The filtrate was mixed with a logarithmic-phase (OD_600_ = 0.6–0.8) Vp bacterial solution at a specified ratio in LB medium containing 3% NaCl and cultured overnight with shaking at 37°C. The culture solution was centrifuged at 8,000 rpm for 10 min, and the supernatant was collected and filtered once more. Then, 10 μL of the obtained filtrate was spotted onto an LB agar plate inoculated with host bacteria and cultured at 37°C for 6 to 8 h to observe plaque formation. The plaques were picked, soaked in SM buffer, and left to stand at 4°C overnight. Next, phages were cultured using the double-layer agar method (29). Individual phage plaques were picked and immersed in SM buffer, then left to stand overnight at 4°C. This purification process was repeated three times until phage plaques of uniform morphology were obtained.

### Assay 2—phage titer assay

Phage titers were determined using the double-layer agar method. Briefly, the phage suspension was serially diluted, and 100 μL of each dilution was mixed with an equal volume of host bacterial culture. The mixture was added to molten LB semi-solid agar (previously boiled and cooled to 50°C–55°C), gently vortexed, and immediately poured onto LB agar base plates. After solidification, the plates were incubated inverted at 37°C for 8–12 h. Plaques formed on the double-layer plates were counted. The phage titer (PFU/mL) = (number of plaques × dilution factor) / volume of inoculum (mL).

### Assay 3—phage Enrichment

A high-titer phage stock was prepared using the liquid co-culture method (30). Briefly, host bacteria were cultured to logarithmic phase, after which equal volumes of bacterial culture and phage suspension were added to LB liquid medium supplemented with 3% NaCl. The mixture was incubated at 37°C with shaking at 180 rpm for approximately 5 h. Following incubation, the culture was centrifuged at 8,000 rpm for 10 min to remove bacterial debris. The supernatant was then filtered through a 0.22 μm membrane filter to eliminate residual bacteria, and the resulting phage filtrate was stored at 4°C for subsequent experiments. This filtration step ensures the removal of bacteria from the phage lysate without the need for chemical treatment.

### Assay 4—host range determination

The host range of the phage was determined using the spot test method (31). The test strains included 15 *Vibrio* strains: *Vibrio parahaemolyticus* (Vp07, Vp44, Vp53, Vp82, Vp94, Vp96, Vp101), *Vibrio alginolyticus* (Va03, Va67, Va147), *Vibrio harveyi* (Vh01, Vh07), *Vibrio vulnificus* (Vv01, Vv02), and *Vibrio mimicus* (Vm01). All strains were confirmed as MDR *Vibrio* species through preliminary antibiotic susceptibility testing (32). The logarithmic-phase bacteria were mixed with warm LB medium containing 0.5% agar and poured onto an agar plate. After solidification, 10 μL of phage solution was spotted onto each bacterial lawn and cultured overnight at 37°C. The presence of clear plaques indicates a susceptible strain.

### Assay 5—observation by transmission electron microscopy

The phage suspension was concentrated to a titer of 10^9^ to 10^10^ PFU/mL. Then, 5 μL of the suspension was placed onto a copper grid and subjected to negative staining with 2% (w/v) phosphotungstic acid (33). The morphological structure was subsequently observed using a Hitachi H-7650 transmission electron microscope.

### Assay 6—optimal multiplicity of infection (MOI)

To determine the MOI of the phages, they were mixed with the host bacterial suspension in the logarithmic phase at MOI of 0.001, 0.01, 0.1, 1, 10, and 100. The mixture was incubated at 37°C with shaking for 5 h, followed by centrifugation at 8,000 rpm for 10 min. The supernatant was collected for phage titer determination. The MOI corresponding to the highest titer was considered the optimal multiplicity of infection.

### Assay 7—one-step growth curve

One-step growth curves were performed to determine the latent period and burst size of the phage, following the protocol described by Hyman et al. (34). The phage and host bacteria were mixed at the optimal MOI and incubated at 37°C for 15 min to allow full adsorption of the phages to the bacterial surface. The mixture was then centrifuged at 12,000 rpm for 10 min to remove unabsorbed phages. The precipitate was resuspended in 5 mL of LB liquid medium and cultured with shaking at 37°C. Samples were collected every 10 min for the first 90 min, followed by collections every 30 min for a total duration of 180 min. The samples were centrifuged at 12,000 rpm for 2 min, and the supernatant was harvested to determine the phage titer.

### Assay 8—thermal stability and pH stability

To assess thermal stability, the phage suspension was incubated in a water bath at temperatures ranging from 30°C to 80°C for 1 h, after which the phage titer was determined following serial dilution.

For pH tolerance, the pH of the SM buffer was adjusted to a range of 2 to 13 using NaOH or HCl solution. After sterilization by filtration, the buffer was mixed with an equal volume of phage suspension and incubated at 37°C for 1 h, followed by serial dilution and phage titer determination.

### Assay 9—phage lytic activity

The lytic activity of phage Φ170 against host bacteria was assessed using a liquid culture assay (35). Briefly, 100 µL of host bacterial culture in logarithmic phase (~10^8^ CFU/mL) was mixed with 100 µL of phage suspension (~10^7^ PFU/mL) in each well of a sterile 96-well plate. For the control group, an equal volume of LB medium 3% NaCl was added instead of phage suspension. The plate was placed in a microplate reader and incubated at 37°C with shaking every 15 min. Bacterial growth was monitored by measuring the optical density (OD_600_) at hourly intervals. The inhibitory effect of phage on bacterial proliferation was evaluated by comparing OD_600_ values between the experimental and control groups. Each condition was tested in triplicate, and the entire experiment was repeated three times. Further, the number of viable bacteria following phage-mediated lysis of the host was determined. Equal volumes of host bacteria and phage were inoculated into 5 mL of 3% NaCl LB medium. Samples were collected at hourly intervals, serially diluted, and subsequently plated onto TCBS agar plates. The plates were incubated at 37°C for 12 h, and the number of surviving colonies was counted to evaluate the lysis kinetics. Total viable colonies (CFU/mL) = number of colonies × dilution factor × 10.

### Assay 10—evaluation of phage-antibiotic combination

The minimum inhibitory concentration (MIC) of doxycycline and the synergistic activity of the combination with phage were tested by the microbroth dilution method (36). Doxycycline hydrochloride was dissolved in PBS buffer to prepare a stock solution at a concentration of 2,560 μg/mL. The stock solution was then diluted 10-fold in Mueller-Hinton (MH) broth to 256 μg/mL, followed by twofold serial dilution in a sterile 96-well plate to achieve final concentrations ranging from 128 to 0.25 μg/mL. The positive control well (0 μg/mL) contained an equal volume of MH broth without doxycycline. All assays were performed in triplicate across three independent experiments. Detailed experimental groupings and volumes are presented in Table 1.

**TABLE 1:** Grouping design for MIC assays of doxycycline alone and in combination with phage

Group	Grouping design
Positive control	50 μL 10^5^ Vp94 + 150 µL MH
Vp94 + DOX	50 μL 10^5^ Vp94 + 100 µL DOX + 50 µL MH
Vp94 + DOX + Φ170	50 μL 10^5^ Vp94 + 100 µL DOX + 50 µL 10^7^ Φ170

The combined bacteriostatic effect of doxycycline and phage was determined. In a 96-well plate, doxycycline was added at concentrations corresponding to the MIC and half of the MIC, along with phage suspension and bacterial suspension. The control groups consisted of phage alone, doxycycline alone and a mixture of culture medium with host bacteria only. The bacterial density (OD_600_) was measured every hour using a multimode reader at 37°C, with shaking every 15 min, to evaluate the synergistic antimicrobial effect. All assays were performed in triplicate across three independent experiments. Detailed experimental groupings and volumes are presented in Table 2.

**TABLE 2:** Grouping design for assessing the combined inhibitory effect of phage and doxycycline

Group	Grouping design
Positive control	50 μL 10^5^ Vp94 + 150 µL MH
Vp94 + Φ170	50 μL 10^5^ Vp94 + 50 µL 10^7^ Φ170 + 100 µL MH
Vp94 + DOX (2 μg/mL)	50 μL 10^5^ Vp94 + 100 µL DOX + 50 µL MH
Vp94 + DOX (2 μg/mL) + Φ170	50 μL 10^5^ Vp94 + 100 µL DOX + 50 µL 10^7^ Φ170
Vp94 + DOX (1 μg/mL)	50 μL 10^5^ Vp94 + 100 µL DOX + 50 µL MH
Vp94 + DOX (1 μg/mL) + Φ170	50 μL 10^5^ Vp94 + 100 µL DOX + 50 µL 10^7^ Φ170

### Zebrafish care and maintenance

Adult zebrafish (*Danio rerio*) used in this study were 5–6 months old, measuring approximately 2.5–3 cm in length, and were purchased from YaoShunYu biotechnology, Nanjing, China. All zebrafish were housed individually in the Laboratory Animal Center of Nanjing Agricultural University (Permit number SYXK (SU) 2011-0036). Zebrafish were reared following Westerfield (37) in large polycarbonate tanks (10 L; 35 cm × 25 cm × 18 cm). For experimental grouping, fish were randomly allocated to smaller polycarbonate tanks (3 L; 26 cm × 16 cm × 12 cm). The rearing system was equipped with an air pump, a heater to maintain water temperature at 28°C, and a filtration unit. Prior to experimentation, the zebrafish were reared in the laboratory for 2 weeks to acclimate to the environment before being used in infection and treatment trials.

### Zebrafish euthanasia

Zebrafish euthanasia was performed using the rapid cooling method, as described by Wilson (38) and Wallace et al. (39). Adult zebrafish were fully immersed in an ice-water mixture (0°C–4°C) for at least 10 min, until complete cessation of opercular movement was observed. Following removal from the ice-water bath, spinal transection was performed as a secondary confirmatory measure to ensure death. All surviving zebrafish at the conclusion of the *in vivo* experiments were euthanized following the procedure described above. Carcasses were sealed, stored at −20°C, and subsequently transported to the university’s institutional biohazard waste facility for safe disposal.

### Pre-experimental detection

Prior to experimentation, zebrafish and water samples from the rearing system were tested for Vp and phage. For pathogen detection, zebrafish were randomly selected from the reserve stock and euthanized; the intestine, liver, and gill tissues were collected and inoculated onto TCBS agar plates, followed by incubation at 37°C for 12–18 h. Meanwhile, water samples from different rearing tanks were spread onto TCBS agar and incubated under identical conditions to observe the presence of characteristic Vp colonies. For phage detection, the same tissue and water samples were subjected to phage isolation using the double-layer agar method with Vp94 as the host, and the plates were examined for the presence of plaques.

### Evaluation of the effect of phages in zebrafish

To evaluate the preventive effect of phage immersion, zebrafish were divided into four groups, with detailed experimental groupings and treatment regimens provided in Table 3. Phage prophylaxis was administered by adding undiluted phage stock directly to the culture water at a 1:1,000 dilution, yielding a final concentration of 10^7^ PFU/mL. Fish were continuously monitored for 72 h, and survival rates were recorded for each group. Each experimental group consisted of 3o fish, and the experiment was independently repeated three times.

**TABLE 3:** Grouping and treatment protocols for the phage prophylaxis experiment

Group	Treatment protocol	Number of fish
PBS control	Intraperitoneal injection of 10 μL PBS	45
Φ170 control	Phage added to tank water (final concentration 10^7^ PFU/mL) for 12 h pretreatment, followed by intraperitoneal injection of 10 μL PBS	45
Vp94 infection	Intraperitoneal injection of 10 μL Vp94 (final concentration 10^5^ CFU/mL)	50
Φ170 prophylaxis	Phage added to tank water (final concentration 10^7^ PFU/mL) for 12 h pretreatment, followed by intraperitoneal injection of 10 μL Vp94 (final concentration 10^5^ CFU/mL)	50

The phage and doxycycline combination treatment experiment was divided into six groups. The Vp94 infection and PBS control groups were the same as those in the prevention experiment, and the detailed grouping and treatment protocols are shown in Table 4. Fish were continuously monitored for 72 h, and survival rates were recorded for each group. Each experimental group consisted of 30 fish, and the experiment was independently repeated three times. Notably, a parallel cohort design was adopted to evaluate *in vivo* parameters in zebrafish (e.g., bacterial load and histopathology). Independent cohorts of 15–20 zebrafish each were established under identical experimental conditions specifically for scheduled sampling. These cohorts did not overlap with those used for survival observation.

**TABLE 4:** Grouping and treatment protocols for the phage-doxycycline combination therapy experiment

Group	Treatment protocol	Number of fish
PBS control	Intraperitoneal injection of 10 μL PBS	45
Φ170 control	Intraperitoneal injection of 10 μL PBS, followed 1 h later by addition of phage to tank water (final concentration 10^7^ PFU/mL)	45
Vp94 infection	Intraperitoneal injection of 10μL Vp94 (final concentration 10^5^ CFU/mL)	50
Φ170 treatment	Phage added to tank water (final concentration 10^7^ PFU/mL) at 1 h post-infection	50
DOX treatment	Doxycycline added to tank water (final concentration 4 μg/mL) at 1 h post-infection	50
Φ170 + DOX treatment	Both phage (final concentration 10^7^ PFU/mL) and doxycycline (final concentration 4 μg/mL) added to tank water at 1 h post-infection	50

### Real-time quantitative PCR

The expression levels of pro-inflammatory cytokines (IL-1β, IL-6, and TNF-α) were measured in zebrafish by real-time quantitative PCR (RT-qPCR). At 4 h post-infection, three zebrafish from each group were randomly selected, humanely euthanized, and their liver and intestinal tissues were immediately collected. Total RNA was extracted using the Trizol method (40), followed by reverse transcription into cDNA with the HiScriptIIRT Kit (Vazyme, R222-01). The transcriptional level of the target gene was detected by the CT method based on the expression level of the housekeeping gene β-actin. The primers of RT-PCR are listed below: β-actin (F: ATGGATGATGAAATTGCCG, R: TGACACCCTGATGTCTGGGG), IL1-β(F: ATCCAAACGGATACGACCAG, R: TCGGTGTCTTTCCTGTCCAT), IL-6(F: TCAGAGACGAGCAGTTTGAG, R: CTTTATACCACGTCAGGACGC), and TNF-α(F: TTGGATGTTGAAGAAGGAGAGT, R: TTATGGAGCGTGAAGCAGAC) (41).

### Bacterial clearance and phage quantification

At 4 and 12 h post-infection, three zebrafish were randomly selected from each group, euthanized and dissected, and their liver and intestinal tissues were collected. After homogenization, the samples were serially diluted and plated onto TCBS agar and incubated at 37°C overnight. The bacterial and phage titers in both the liver and intestine of each group were determined by plate counting and the double agar layer method.

### Histopathology of zebrafish

At 12 h post-infection, three zebrafish per group were randomly selected and humanely euthanized. Liver and intestinal tissues were then harvested and immediately fixed in 4% paraformaldehyde for 24 h. After fixation, the tissues were dehydrated through a graded series of ethanol and xylene. The tissues were then embedded in paraffin and sectioned into 5-µm thick slices. The sections were stained with hematoxylin and eosin (H&E) for histopathological analysis. Tissue morphology was observed under an optical microscope (LEICA DM500, Germany).

### Statistics

The data in this study were representative of at least three independent experiments. All data were statistically analyzed using GraphPad Prism 7.0 software. The *t*-test was used to determine the significance of differences between two groups, and one-way analysis of variance (ANOVA) was used to assess the significance of differences among multiple groups, with post-hoc analyses (Tukey’s or Bonferroni’s test) applied as appropriate. Survival data were analyzed using the Kaplan-Meier estimator, and differences in survival distributions were compared using the log-rank (Mantel-Cox) test. All data were represented as mean ± standard deviation (mean ± SD). Differences were considered statistically significant when *P* < 0.05, where *P < 0.05, **P < 0.01, ***P < 0.001, ****P < 0.0001, ns, no significant difference.

## RESULTS

### The morphology and host range of phage Φ170

**Fig 1:**
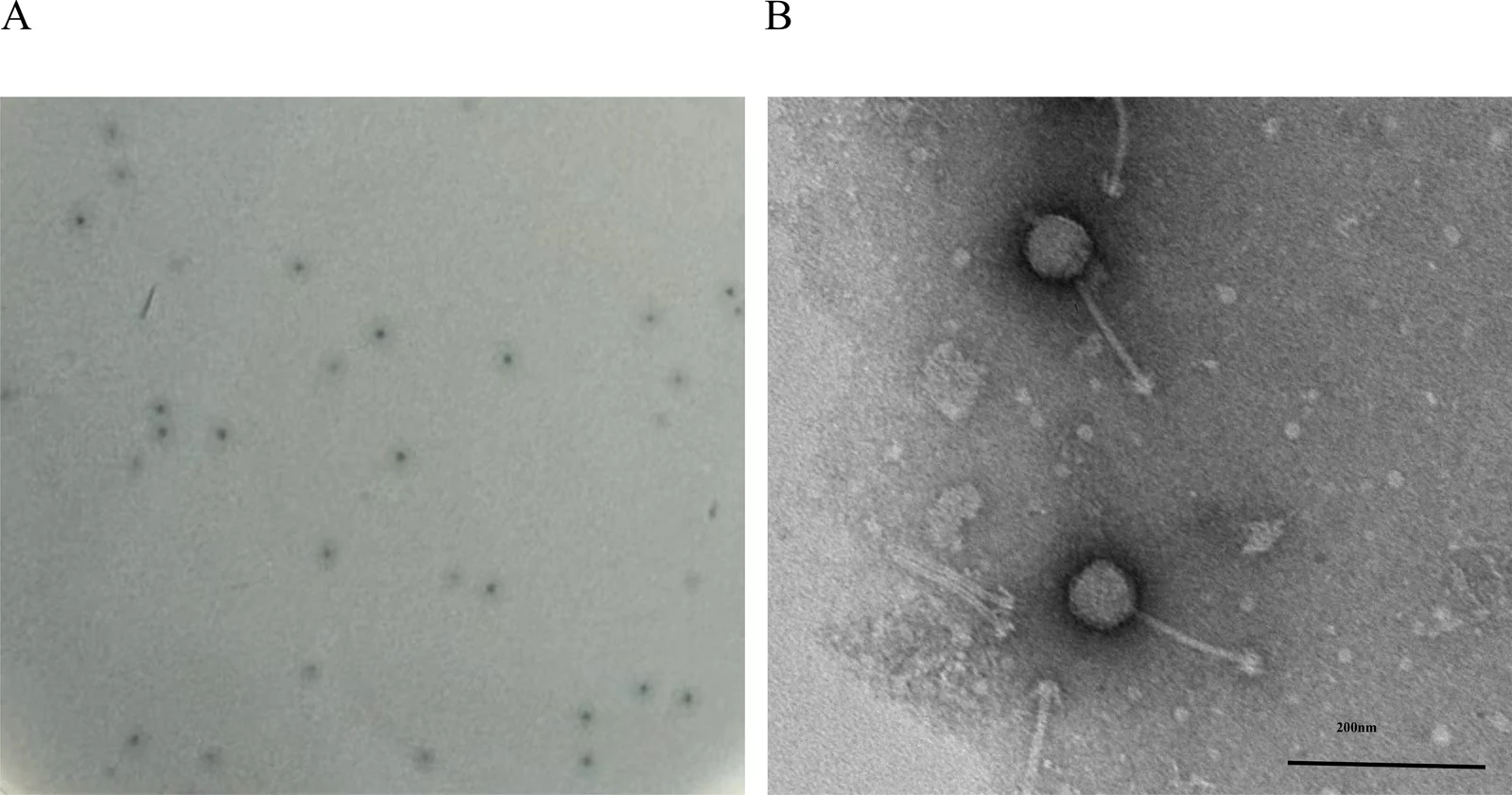
Morphology of phage Φ170. (**A**) Plaque morphology of phage Φ170 on double-layer agar plates. (**B**) Transmission electron micrograph showing the morphology of phage Φ170, with the scale bar representing 200 nm.

Phage Φ170 was isolated from sewage collected at a shrimp farm in Jiangsu Province, China. After incubation on double-layer agar plates at 37°C for 6 h, uniform circular plaques approximately 1 mm in diameter were observed, characterized by diffuse edges and surrounding halos (Fig. 1A). Transmission electron microscopy (TEM) analysis revealed that the phage possessed a hexagonal head approximately 56 nm in diameter and a long, non-contractile tail measuring about 120 nm in length (Fig. 1B). Based on these morphological features, Φ170 was classified as a member of the family *Siphoviridae*. The lysis spectrum of Φ170 against 15 MDR *Vibrio* strains was assessed using a spot assay. As shown in Table 5, Φ170 lysed five of the nine tested Vp strains but exhibited no lytic activity against other *Vibrio* species, indicating a high degree of strain specificity.

**TABLE 5:** Host range of phage Φ170^*a*^

Strains	Species	Drug resistance	Lysis
Vp53	*V. parahaemolyticus*	AMP, PCN, ENR, STR, GEN, AMK	+
Vp82	*V. parahaemolyticus*	AMP, PCN, ENR, STR, GEN, AMK	+
Vp94^*b*^	*V. parahaemolyticus*	AMP, PCN, ENR, STR, GEN, AMK, TET	+
Vp96	*V. parahaemolyticus*	AMP, PCN, CTX, STR, TET, DOX	+
Vp101	*V. parahaemolyticus*	AMP, PCN, ENR, STR, GEN, AMK	+
Vp07	*V. parahaemolyticus*	AMP, ENR, STR, GEN	−
Vp44	*V. parahaemolyticus*	AMP, PCN, ENR, STR, GEN	−
Vp109	*V. parahaemolyticus*	AMP, ENR, STR, GEN, AMK	−
Vp133	*V. parahaemolyticus*	ENR, STR, GEN, AMK	−
Va03	*V. alginolyticus*	STR, PCN, GEN, AMK	−
Va67	*V. alginolyticus*	AMP, PCN, AMK, TET, DOX	−
Va147	*V. alginolyticus*	AMP, PCN, STR, GEN	−
Vh01	*V. harveyi*	ENR, PCN, STR, AMK, TET	−
Vh07	*V. harveyi*	AMP, PCN, ENR, STR, AMK	−
Vv01	*V. vulnificus*	AMP, PCN, TET	−
Vv02	*V. vulnificus*	ENR, STR, GEN, AMK	−
Vm01	*V. mimicus*	AMP, PCN, CTX, STR	−

^*a*^ AMP, ampicillin; PCN, penicillin; ENR, enrofloxacin; CTX, cefotaxime; STR, streptomycin; GEN, gentamicin; AMK, amikacin; TET, tetracycline; DOX, doxycycline. +: clear lysis zone,  −: no lysis zone.

^*b*^ Host bacteria.

### Optimal MOI and one-step growth curve

**Fig 2:**
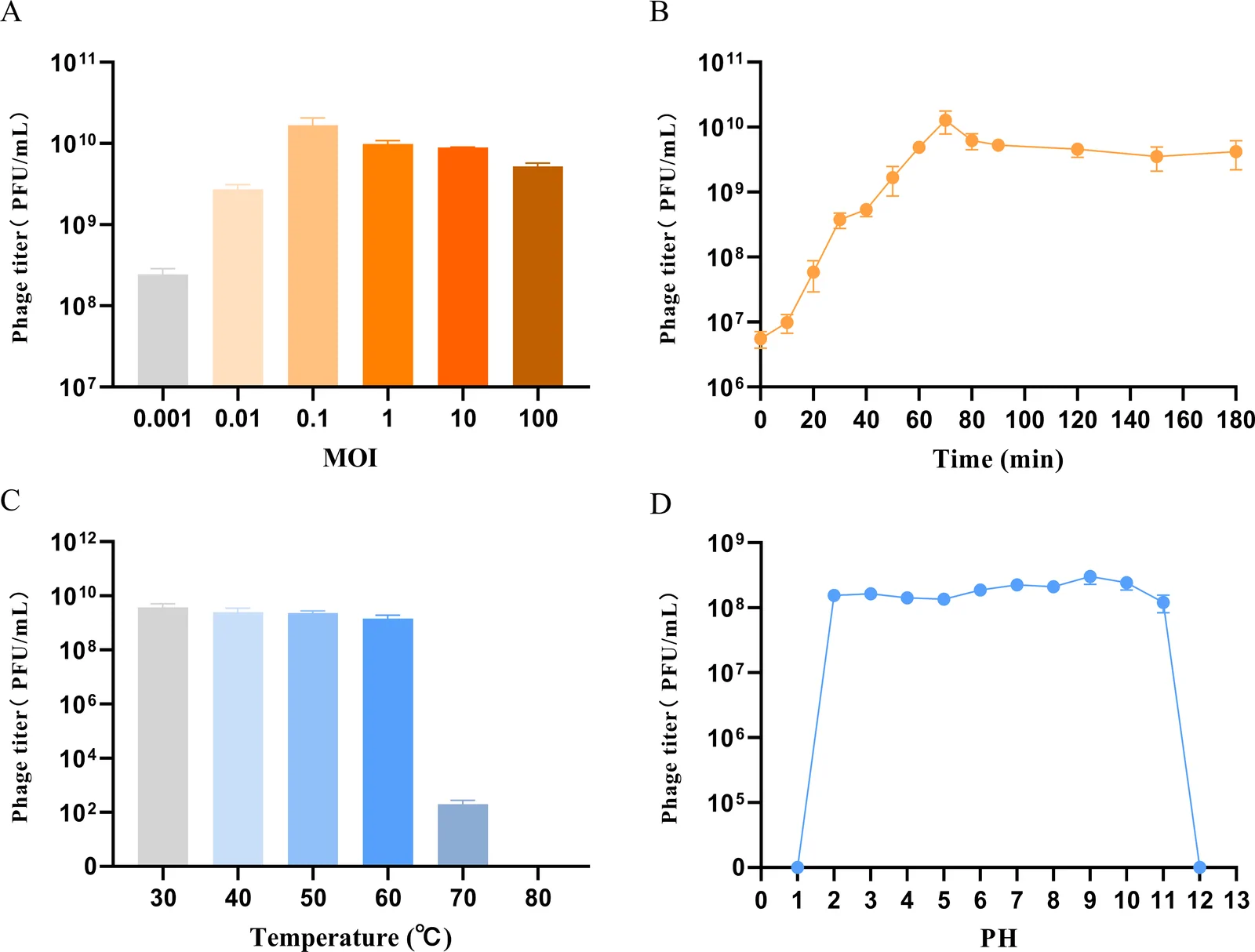
Biological characteristics of phage Φ170. (**A**) The optimal MOI of Φ170. (**B**) One-step growth curve of Φ170. (**C**) Thermal stability of Φ170. (**D**) pH stability of Φ170. The results were presented as the mean ± standard deviation of three independent experiments.

To determine the optimal MOI, phage yields were measured at different MOI values. The results showed that the phage titer reached a maximum of approximately 10^10^ PFU/mL at an MOI of 0.1 (Fig. 2A). One-step growth curve analysis indicated that Φ170 had a latent period of approximately 10 min, followed by a burst phase lasting about 60 min, with an average burst size of 426 PFU/cell (Fig. 2B).

### Thermal and pH stability

To evaluate the stability of Φ170, phage suspensions were exposed to different temperatures and pH conditions, and the resulting phage titers were determined. The results showed that Φ170 remained stable at temperatures ranging from 30°C to 60°C (Fig. 2C). However, the titer decreased by approximately six orders of magnitude after incubation at 70°C for 1 h, and complete inactivation occurred at 80°C (Fig. 2C). Regarding pH stability, Φ170 remained stable within a pH range of 2 to 11 but was completely inactivated under extreme conditions of pH 1 and 12 (Fig. 2D).

### Phage exhibit *in vitro* lytic activity and synergize with antibiotics to inhibit bacterial growth

**Fig 3:**
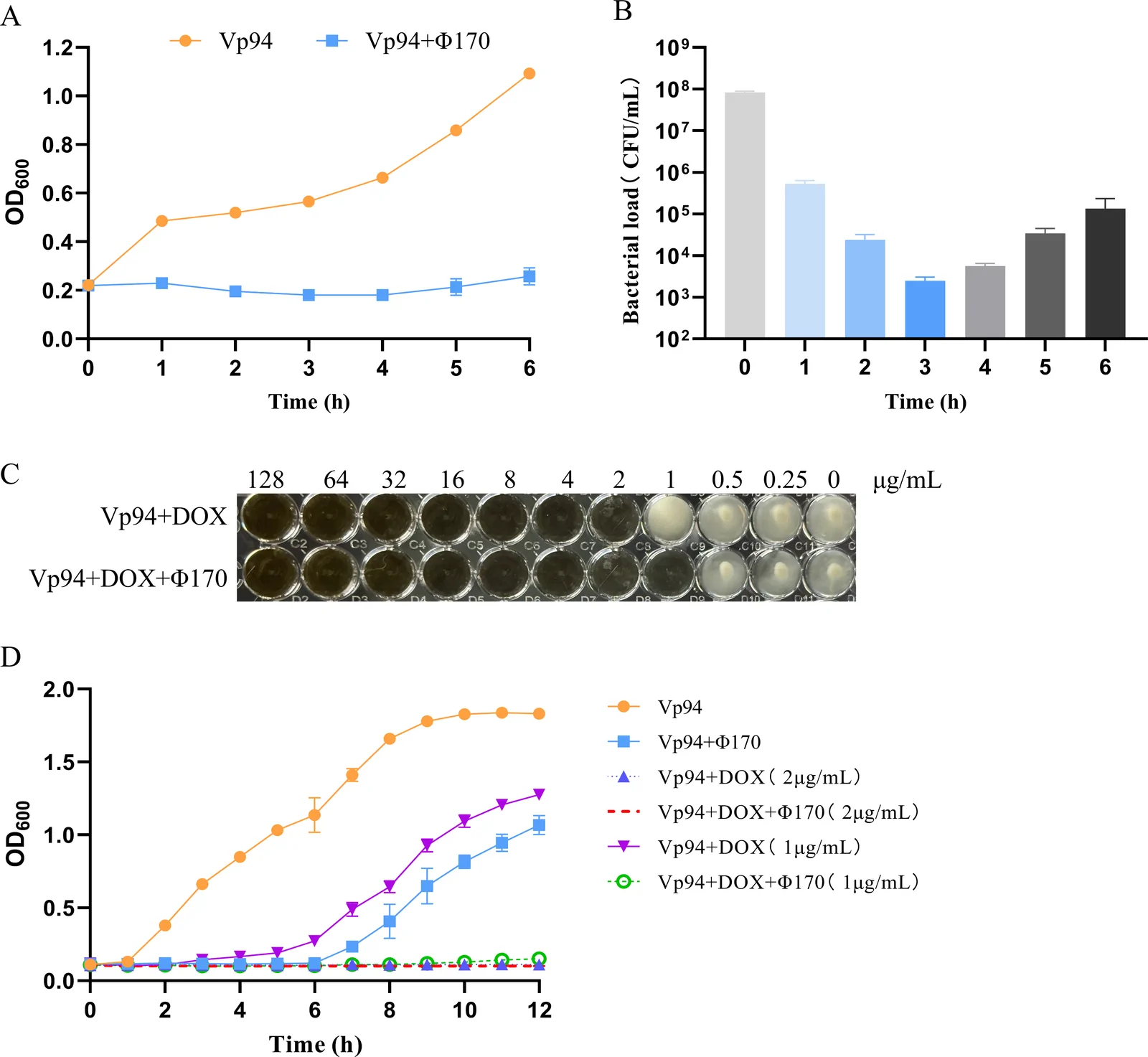
*In vitro* lytic activity of phage Φ170 and its synergistic antibacterial effect with antibiotics. (**A**) Growth inhibition curve of phage Φ170. (**B**) The lysis effect of Φ170 on host bacteria. (**C**) MIC of doxycycline and its combination with Φ170. (**D**) Antibacterial effect of the Φ170-doxycycline combination. At MIC and 1/2 MIC concentrations, the effects of doxycycline and the Φ170-doxycycline combination on Vp94 growth were determined. The results are presented as the mean ± standard deviation of three independent experiments.

*In vitro* lysis kinetics analysis demonstrated that, compared with the control group, phage Φ170 effectively lysed the host bacterium Vp94, resulting in significantly inhibiting bacterial growth (Fig. 3A). The inhibitory effect was most pronounced at 3–4 h post-infection (Fig. 3A). Consistently, viable cell counting showed that the bacterial burden of Vp94 reached its lowest level at 3–4 h (Fig. 3B). These results indicated that Φ170 exhibited rapid and efficient lytic activity against Vp94. Previous experiments indicated that Vp94 was multidrug resistant and sensitive only to doxycycline and chloramphenicol (Table S1). Since chloramphenicol is prohibited in aquaculture, doxycycline was selected for combination therapy experiments with the phage in this study. Microbroth dilution assays showed that the MIC of doxycycline against Vp94 was 2 μg/mL, which decreased to 1 μg/mL when combined with phage Φ170 (Fig. 3C), indicating a synergistic antibacterial effect between the phage and doxycycline. Further analysis demonstrated that at 2 μg/mL doxycycline, a concentration that completely inhibited Vp94 growth, co-administration with Φ170 did not further enhance bactericidal effect (Fig. 3D), owing to the absence of residual bacteria required for phage proliferation. In contrast, under a subinhibitory concentration of doxycycline (1 μg/mL), Vp94 exhibited regrowth after 5 h, whereas combined treatment with Φ170 effectively suppressed bacteria proliferation (Fig. 3D). These results indicated that the combination of doxycycline and Φ170 exerted a significantly stronger antibacterial effect than either treatment alone, and demonstrating synergistic activity *in vitro*.

### Prophylactic administration of phage Φ170 reduced *Vibrio parahaemolyticus* infection and disease burden in zebrafish

**Fig 4:**
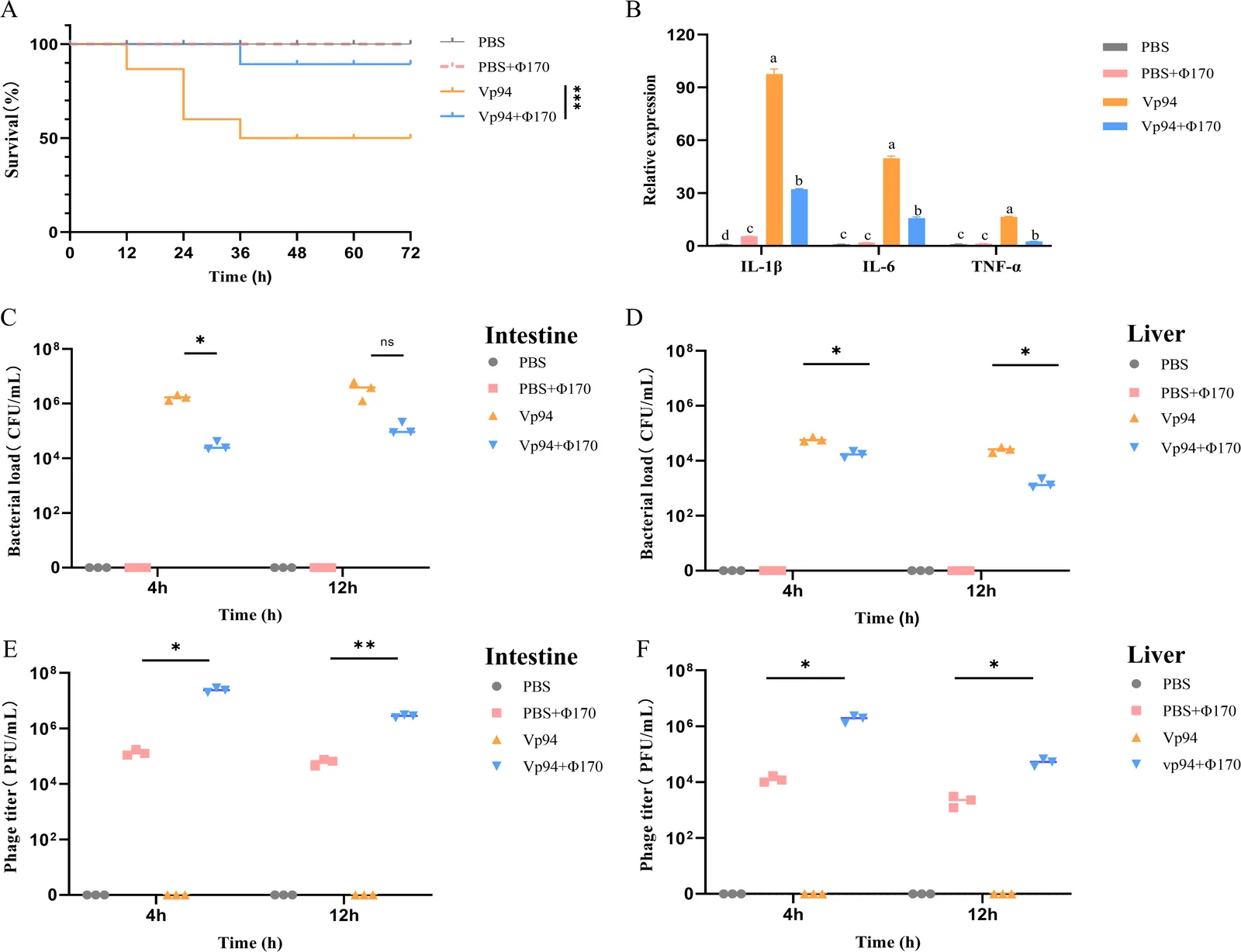
Phage Φ170 effectively prevented *Vibrio parahaemolyticus* infection in zebrafish. (**A**) Survival curve of zebrafish. Survival was observed and recorded for each group of zebrafish over a 72h period (*n* = 30). (**B**) Expression levels of IL-1β, IL-6, and TNF-α in zebrafish (*n* = 3). The same letter indicates no significant difference between groups, different letters indicate a significant difference (*P* < 0.05). (**C**) bacterial load in the intestine; (**D**) bacterial load in the liver; (**E**) phage titer in the intestine; (**F**) phage titer in the liver; (*n* = 3). The results are presented as the mean ± standard deviation of three repeated experiments. ***P < 0.001, **P < 0.01, *P < 0.05, ns, no significant difference.

Prior to the start of the experiments, the absence of Vp and phage Φ170 were confirmed both in the zebrafish and the housing water. To assess the prophylactic effect of phage Φ170 *in vivo*, phages were added to the rearing water (~10^7^ PFU/mL) 12 h prior to zebrafish infection with Vp94. Survival curve analysis showed that zebrafish in the infection group had a 50% survival rate, whereas those in the prophylaxis group exhibited a significantly higher survival rate of 90% (Fig. 4A). This result indicated that Φ170 effectively protected zebrafish from Vp94 infection. No mortality was observed in the PBS control and phage-only treatment groups within 72 h. Moreover, compared with the PBS control, the expression levels of pro-inflammatory cytokines were not significantly elevated in the phage-only group (Fig. 4B). These results indicated that phage Φ170 is non-toxic to zebrafish. Compared with the infection group, the expression levels of pro-inflammatory cytokines were significantly reduced in the liver and intestine of zebrafish in the prophylaxis group (Fig. 4B). Further analysis of tissue bacterial load and phage titers showed that at 4 h post-infection, bacterial burdens in both the intestine and liver of the prophylaxis group were reduced by approximately 1–2 logs compared with the infection group (Fig. 4C and D). Over time, the protective effect of Φ170 gradually diminished; by 12 h, although intestinal bacterial loads remained lower than those in the infection group, the difference was no longer statistically significant (Fig. 4C). In contrast, Φ170 continued to exert a significant protective effect in the liver at this time point (Fig. 4D). Notably, phage titers increased by approximately 1–2 logs at both 4 and 12 h post-infection (Fig. 4E and F). These findings indicated that Φ170 could effectively propagate *in vivo* and reduce pathogen burden, thereby providing a prophylactic effect.

### Synergistic phage–antibiotic treatment effectively reduced *Vibrio parahaemolyticus* infection in zebrafish

**Fig 5:**
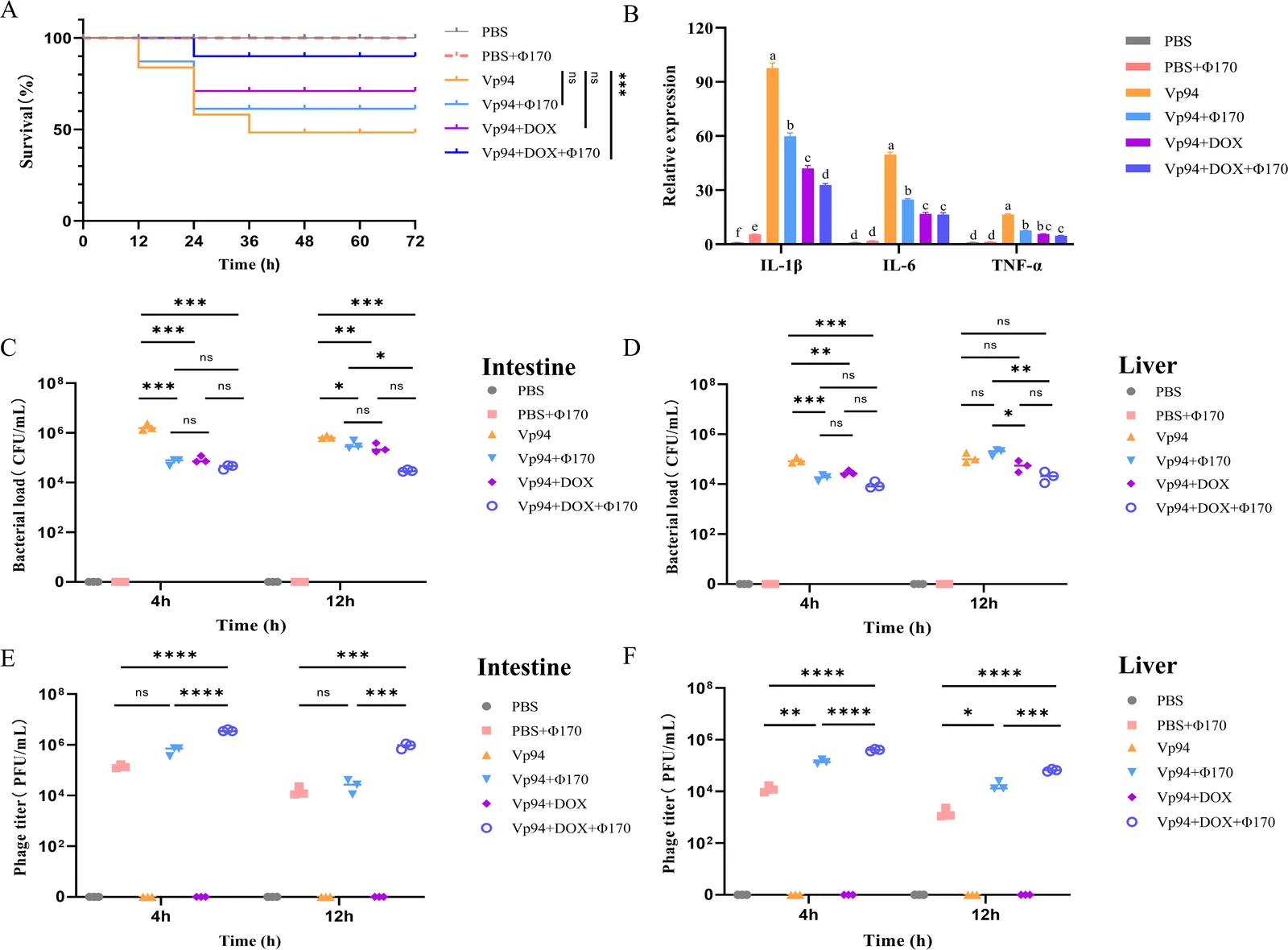
The combination of phage Φ170 and antibiotics synergistically and effectively treated *Vibrio parahaemolyticus* infection in zebrafish. (**A**) Survival curve of zebrafish. Survival was observed and recorded for each group of zebrafish over a 72 h period (*n* = 30). (**B**) Expression levels of IL-1β, IL-6, and TNF-α in zebrafish (*n* = 3). The same letter indicates no significant difference between groups, different letters indicate a significant difference (*P* < 0.05). (**C**) bacterial load in the intestine; (**D**) bacterial load in the liver; (**E**) phage titer in the intestine; (**F**) phage titer in the liver (*n* = 3). The results are presented as the mean ± standard deviation of three repeated experiments. SD. ****P < 0.0001, ***P < 0.001, **P < 0.01, *P < 0.05, *ns*, no significant difference.

To evaluate the therapeutic efficacy of phage Φ170 in the zebrafish model, infected zebrafish were treated 1 h post-infection with phage Φ170 alone, doxycycline alone, or a combination of phage and antibiotic. In preliminary *in vitro* assays, the MIC of doxycycline against Vp94 was determined to be 2 μg/mL. However, in the *in vivo* pre-experiment with zebrafish, this concentration did not provide noticeable therapeutic efficacy, as the survival rate was comparable to that of the infected group (Fig. S1). Therefore, a concentration of 2 × MIC (4 μg/mL) doxycycline was used for subsequent *in vivo* treatment. Survival analysis showed that, compared with the infection group, survival rates increased by 13.33%, 23.33%, and 43.33% in the phage-only, doxycycline-only, and combined treatment groups, respectively (Fig. 5A). Notably, the combined treatment group achieved a survival rate of 90%, which was significantly higher than that observed in either single-treatment group (Fig. 5A). These results indicated that the combination of Φ170 and doxycycline could more effectively rescue zebrafish from Vp94 infection.

Analysis of inflammatory cytokine expression revealed that all treatment groups exhibited lower levels of the three pro-inflammatory cytokines compared with the infection group (Fig. 5B). Among the treatment groups, the combined therapy group showed the greatest reduction in IL-1β expression, whereas no significant differences in IL-6 and TNF-α levels were observed among the treatment groups (Fig. 5B). These results indicated that all three treatment regimens attenuate the pro-inflammatory response induced by Vp94, with the combined therapy producing the most pronounced effect. Further analysis of bacterial loads and phage titers in intestinal and liver tissues showed that at 4 h post-infection, all treatment groups significantly reduced tissue bacterial burdens (Fig. 5C and D). By 12 h, all treatment groups maintained an antibacterial trend in the intestine, with the combination group exhibiting the most pronounced effect achieving the lowest bacterial load and outperforming phage monotherapy (Fig. 5C). In liver tissue, although bacterial loads rebounded by 12 h and the effects of individual treatment groups were no longer statistically significant, the combination group still demonstrated significantly better bacterial reduction than phage alone, suggesting limited efficacy of phage monotherapy at later stages and the superiority of combination therapy. Phage titer measurements showed that the combination group maintained titers of approximately 10^6^ PFU/mL in both intestinal and liver tissues, significantly higher than those observed in the phage-alone group (Fig. 5E and F). Collectively, these results indicate that the combination of Φ170 and doxycycline promotes sustained phage proliferation *in vivo*, leading to significantly reduced bacterial loads during the early stage of infection and conferring a synergistic therapeutic effect.

### Histopathology of zebrafish tissues

**Fig 6:**
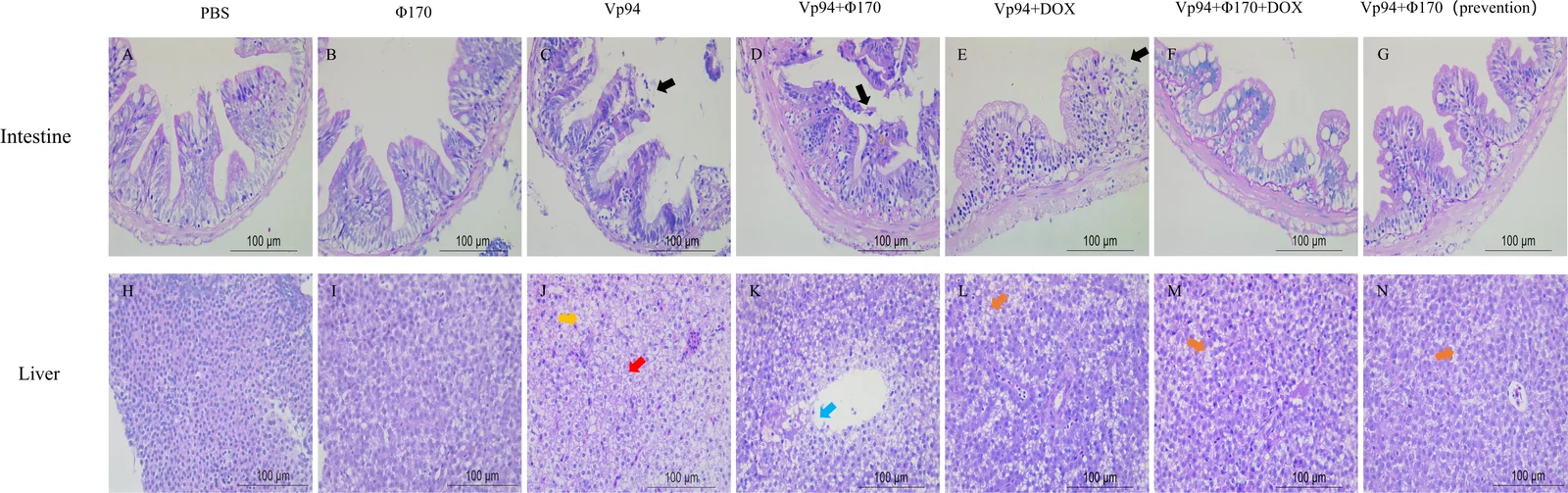
Histopathological features of the intestine and liver tissues in zebrafish from various groups. At 12 h post-infection, intestines (**A–G**) and livers (**H–N**) of zebrafish from each group were collected and fixed in 4% paraformaldehyde for 24 h. Tissue sections were stained with hematoxylin and eosin (H&E) and examined using a light microscope at 40× magnification. Black arrows indicate the detachment of intestinal epithelial cells. Red arrows indicate severe hepatic edema with ballooning degeneration, cytoplasmic loosening, and pale staining. Green arrows point to hepatocyte lysis. Blue arrow: localized liver tissue lysis foci, with surrounding hepatocyte edema, degeneration, and disruption of cell arrangement. Orange arrows indicate mild hydropic degeneration of the liver tissue.

To evaluate the safety and efficacy of phage Φ170 in zebrafish, intestinal and liver tissues from each group were collected 12 h post-infection, stained with hematoxylin and eosin (H&E), and examined for histopathological changes. The results showed no significant pathological alterations in the organs of the PBS and phage-only control groups, with tissue structure appearing normal. In the infected group, the intestinal tissue exhibited mucosal epithelial cell shedding (Fig. 6C). The intestinal lesions in the phage-only group were similar to those in the infected group (Fig. 6D). Treatment with doxycycline alone alleviated mucosal damage compared with the infected group, (Fig. 6E). Following combined treatment with Φ170 and doxycycline, or prophylactic administration of Φ170, intestinal lesions were significantly improved, approaching the normal state observed in uninfected controls (Fig. 6F and G). Additionally, the liver of the infected group exhibited severe edema, ballooning degeneration, and partial hepatocyte lysis (Fig. 6J). After treatment with Φ170 alone, liver edema was partially alleviated, although localized hepatocyte lysis and disorganized hepatocyte arrangement remained (Fig. 6K). Doxycycline treatment alone effectively reduced edema, while combination therapy and Φ170 prophylaxis resulted in only mild edema, with tissue structure restored to normal and significant improvement in pathological changes (Fig. 6L through N). These results indicated that phage Φ170 was relatively safe in zebrafish and exhibited significant prophylactic effects against Vp94, with enhanced therapeutic efficacy when combined with doxycycline.

## DISCUSSION

Phage therapy is increasingly regarded as a promising effective strategy for combating MDR bacterial infections, owing to its advantages, such as high host specificity, self-replicating capacity, and environmental sustainability. It shows particular promise for the prevention and control of aquatic *vibrios* (42). Despite its potential, the practical application of phage therapy faces significant hurdles, such as narrow host specificity, the development of bacterial resistance to phages, and inconsistent therapeutic outcomes in both *in vitro* and *in vivo* settings.

Morphological analysis revealed that phage Φ170 possesses a long, non-contractile tail, classifying it within the *Siphoviridae* family. The phage tail is an important structure for host recognition, triggering adsorption and the opening of membrane channels (43). Host range analysis demonstrated that Φ170 exhibits high strain specificity, effectively lysing only the tested MDR Vp strains, with no activity against other *Vibrio* species. This narrow host range is attributed to the tail protein’s recognition of specific surface receptors on the bacterial cell (44). Under antibiotic pressure, bacteria can alter their surface receptors, such as downregulating outer membrane protein expression or modifying LPS structure. These changes can impair phage recognition and adsorption, resulting in a narrower and more specific lysis spectrum (45). Although this specificity highlights, to some extent, the precise recognition capability of Φ170 against target MDR bacteria, it also severely limits its direct application value in complex infection scenarios (46). To address this limitation, multiple host range expansion strategies can be pursued in future studies. For example, Φ170 can be combined with other phages with complementary host spectra to construct a phage cocktail (47). Φ170 derivatives with an expanded host range can be generated through serial passage adaptation and directed evolution (48, 49). Furthermore, genetic engineering approaches can be employed to modify the phage tail proteins or receptor-binding proteins, thereby expanding the host range (50, 51).

Phages circumvent conventional antibiotic resistance; bacteria can still evolve specific resistance to phages. These two phenomena are not contradictory: antibiotic resistance refers to bacterial mechanisms that neutralize or evade chemical antimicrobials, while phage resistance arises through bacterial adaptations such as receptor modification, CRISPR-Cas systems, or abortive infection mechanisms, which can occur relatively quickly under selective pressure (52). Therefore, phages remain effective against MDR strains, but their long-term efficacy may be limited by the evolutionary capacity of bacteria to develop phage-specific resistance. Notably, the acquisition of phage resistance often imposes a fitness cost on bacteria, manifesting as reduced growth rate, attenuated virulence, or increased susceptibility to other environmental stresses (53). Such trade-offs offer opportunities for combination strategies to control bacterial infections, including the use of phage-antibiotic combinations or phage cocktails that target distinct receptors on the bacterial surface (54, 55). In this study, we combined phage Φ170 with doxycycline, an antibiotic effective against the host bacteria. The results demonstrated a synergistic bacteriostatic effect *in vitro*. This combination not only delayed the emergence of phage resistance but also enhanced overall bactericidal efficacy, consistent with previous studies on phage-antibiotic synergy (56, 57). The underlying mechanism may involve antibiotics disrupting bacterial metabolism or cell wall integrity, thereby facilitating phage adsorption and proliferation. Further investigation is required to elucidate the precise mechanism. Notably, doxycycline is inherently a bacteriostatic antibiotic and does not directly kill bacteria (58). When its effect disappears, residual pathogens in the host may cause infection relapse, potentially leading to a rebound in zebrafish mortality. However, another possibility is that doxycycline suppresses bacterial proliferation, thereby providing a crucial window of opportunity for the host immune system to mount a more effective clearance of the infection. In this scenario, even if the bacteriostatic effects of doxycycline are lost, it would not negatively impact the survival rate of zebrafish.

The prophylactic potential of phage Φ170 and its combinatorial efficacy with doxycycline were systematically evaluated in a zebrafish infection model, wherein phages were delivered via supplementation of the rearing water. To compensate for phage loss in the aquatic environment and during delivery, and to ensure that a sufficient number of phages reach and act on the pathogens, an MOI of 100 was used for infection (59). The MOI used in this study was established based on *in vitro* experiments; it cannot be directly equated to the *in vivo* MOI. In the fish infection model, the environment is more complex: phages are dispersed in the rearing water, while Vp is injected directly into the fish tissues, with no direct contact between the two. Phages must first diffuse from the water into the fish and then penetrate the tissues to reach the target bacteria; consequently, the actual number of phages reaching the infection site is considerably lower than the amount added to the water. Therefore, although increasing phage concentration may enhance its interaction with the target bacteria, preparing ultra-high-titer phage stocks requires optimized culture conditions and multiple concentration steps, which significantly increase production costs and limit economic feasibility. Future studies should further investigate the distribution and clearance kinetics of phages in zebrafish to achieve effective therapy at lower MOI. In addition, comparing the impact of different administration routes on phage concentrations in target tissues may provide valuable insights for optimizing treatment strategies.

The results from zebrafish studies indicate that while Φ170 effectively prevents Vp infection, its therapeutic effect on established infections is limited, consistent with previous findings (60). We hypothesize that this limitation arises from the rapid progression of Vp infection, which prevents phages administered via immersion from reaching an effective therapeutic concentration in tissues once the infection is established. Additionally, a discrepancy was observed between the *in vitro* MIC of doxycycline (2 μg/mL) and its effective *in vivo* concentration (4 μg/mL). This suggests that under immersion conditions, antibiotic tissue penetration and diffusion may be less efficient than in the ideal *in vitro* environment, requiring higher doses *in vivo* to achieve comparable bacteriostatic effects.

In the combined treatment group, the phage-doxycycline combination not only significantly improved the survival rate of infected zebrafish but also reduced the expression levels of pro-inflammatory cytokines (IL-1β, IL-6, TNF-α) in both the liver and intestine. Notably, the downregulation of IL-1β was especially pronounced, highlighting its critical role in the inflammatory response induced by Vp infection. Further analysis revealed that at 4 h post-infection, bacterial loads in both intestinal and liver tissues were significantly different between the infection group and each treatment group, whereas no significant differences were observed among the treatment groups. At 12 h post-infection, bacterial loads in liver tissue did not differ significantly between the infection group and any of the treatment groups. Nevertheless, in terms of therapeutic outcomes, the combination group improved zebrafish survival rates by 30% and 20% compared with phage monotherapy and doxycycline monotherapy, respectively. This may be attributed to the synergistic effect of the phage-antibiotic combination, which effectively reduced inflammation triggered by bacterial lysis, thereby better preserving host organ function. In addition, phages exert minimal disruption on the normal intestinal microbiota, and the combination strategy may also reduce the required dose. of doxycycline, thereby mitigating its impact on the gut microbiota and enabling the zebrafish to mount a stronger recovery during the post-infection period (61). In addition, the phage titer in the combination group remained higher than that in the phage-only group over 12 h, suggesting that doxycycline may promote Φ170 replication within the host and contribute to the observed synergistic bacteriostatic effect. Histopathological examination corroborated these findings, showing significant alleviation of tissue damage in both the prophylactic and combined treatment groups. Furthermore, the phage-only control group did not exhibit notable inflammatory responses or tissue damage, indicating that Φ170 is non-toxic to zebrafish and exhibits favorable biosafety.

Although this study systematically evaluated the biological characteristics of phage Φ170 and its antibacterial efficacy both *in vitro* and *in vivo*, several limitations remain. The host range of Φ170 is relatively narrow, which limits its potential applications. The molecular mechanisms underlying the interaction between phages and antibiotics in combination therapy are not yet understood. Additionally, the diffusion of phages and antibiotics in complex aquatic environments requires further investigation. In future studies, we aim to isolate and screen phages with distinct receptor recognition profiles, develop broad-spectrum phage cocktails, and explore the mechanisms of phage-antibiotic synergy to improve their practical applicability and effectiveness.

In summary, we isolated and characterized phage Φ170, which exhibits strong lytic activity and stability, effectively combating MDR *Vibrio parahaemolyticus* Vp94 infection. Evaluation of its biological properties, *in vitro* lytic capacity, and prophylactic and therapeutic effects in a zebrafish model demonstrated the potential of Φ170 for use alone or in combination with doxycycline. These findings identify Φ170 as a promising candidate for the development of phage-based approaches to prevent and control aquatic diseases and provide experimental evidence supporting further investigation of phage therapy in aquaculture.

## Data Availability

The relevant data supporting the results of this study can be found in the article, the supplemental material, and the source data. Source data are provided with this paper.

## SUPPLEMENTAL MATERIAL

Supplemental material spectrum.00089-26-s0001.docx Table S1 and Figure S1.

ASM does not own the copyrights to Supplemental Material that may be linked to, or accessed through, an article. The authors have granted ASM a non-exclusive, world-wide license to publish the Supplemental Material files. Please contact the corresponding author directly for reuse.
