# Supplementary material for: Phage Φ170 biocontrol of multidrug-resistant Vibrio parahaemolyticus in a zebrafish model
Source: Microbiol Spectr. 2026 May 26;14(7):e00089-26. doi: 10.1128/spectrum.00089-26 (PMC13340044; doi:10.1128/spectrum.00089-26)
Supplement: Supplemental material — Table S1 and Figure S1. [file spectrum.00089-26-s0001.docx]

**Supplementary information for**

**Phage Φ170 biocontrol of multidrug-resistant Vibrio parahaemolyticus in a zebrafish model**

Wanqiu Lin ^1^, Linlin Ye ^1^, Xuhang Wang ^1^, Jianluan Ren ^1^, Feng Xue ^1, 2^, Jianjun Dai ^1, 2^, Fang Tang ^1, 2, *^

* Corresponding authors: Fang Tang

Email: tfalice@126.com (FT)

**Supporting Figures and Legends**

**Table S1 and Figures S1**

**Supplementary Table S1. Results of antimicrobial susceptibility testing for 15 *Vibrio* strains (S: Susceptible, I: Intermediate, R: Resistant)**

|  | Vp53 | Vp82 | **Vp94** | Vp96 | Vp101 | Vp07 | Vp44 | Vp109 | Vp133 | Va03 | Va67 | Va147 | Vh01 | Vh07 | Vv01 | Vv02 | Vm01 |
| --- | --- | --- | --- | --- | --- | --- | --- | --- | --- | --- | --- | --- | --- | --- | --- | --- | --- |
| PEN | R | R | R | R | R | I | R | I | I | R | R | R | R | R | R | I | R |
| AMP | R | R | R | R | R | R | R | R | S | I | R | R | I | R | R | S | R |
| CAZ | I | I | I | S | S | S | S | S | S | S | S | S | I | S | S | S | R |
| CTX | S | I | I | R | I | S | S | S | S | S | S | S | S | S | I | S | R |
| STR | R | R | R | R | R | R | R | R | R | R | I | R | R | R | I | R | R |
| GEN | R | R | R | I | R | R | R | R | R | R | I | R | I | I | I | R | S |
| AMK | R | R | R | S | R | R | I | R | R | R | R | S | R | R | I | R | S |
| TET | S | S | R | R | S | I | S | S | S | S | R | S | R | S | R | S | S |
| DOX, | S | S | S | R | S | S | S | S | S | S | R | S | S | S | S | S | S |
| CIP | R | R | R | S | R | R | R | R | R | I | S | I | R | R | S | R | S |
| SXT | I | I | I | I | I | R | I | I | S | S | I | I | S | R | I | S | R |
| CHL | S | S | S | S | R | S | S | S | S | S | S | S | S | S | S | S | S |

Abbreviations: PEN, Penicillin G; AMP, Ampicillin; CAZ, Ceftazidime; CTX, Cefotaxime; STR, Streptomycin; GEN, Gentamicin; AMK, Amikacin; TET, Tetracycline; DOX, Doxycycline; CIP, Ciprofloxacin; SXT, Trimethoprim-Sulfamethoxazole; CHL, Chloramphenicol.

**Supplementary Figure S1.**


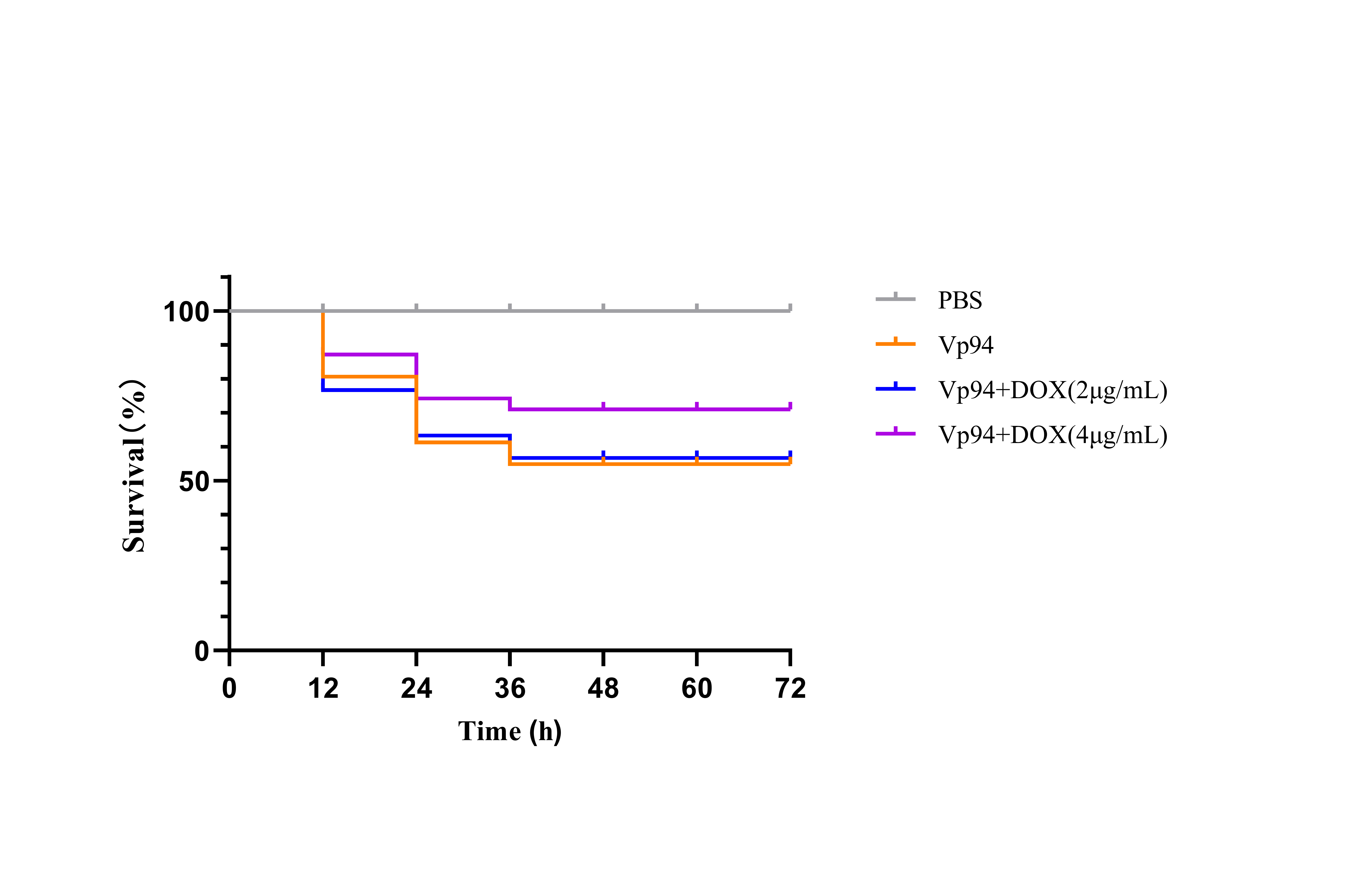


**Fig S1. Survival curve of Vp94-infected zebrafish treated with different concentrations of doxycycline.** Survival was observed and recorded for each group of zebrafish over a 72 h period (n = 10).

The zebrafish were divided into four experimental groups as follows: Infection group: zebrafish were intraperitoneally injected with 10 μL of Vp suspension (~10^5^ CFU/mL). Control group: zebrafish were intraperitoneally injected with 10 μL of PBS. Antibiotic treatment groups: After 1 h post-injection, two different concentrations of the doxycycline (2 μg/mL and 4 μg/mL) were added to the zebrafish culture water. Survival of the zebrafish was monitored and recorded over a period of 72 h. Each group in the preliminary experiment consisted of 10 zebrafish, and the experiment was repeated three times.
